# Static Joint Diseases

**Published:** 1912-03

**Authors:** J. S. Lewis


**Static Joint Diseases. Dr. George Preiser,** Hamburg. A monograph, 278 pages, 272 illustrations. 1911. Verlag von Ferdinand Enke Stuttgart, Germany.

The author makes a very good case for a reasonable classification and treatment of chronic non-inflammatory localized joint conditions with and without bony changes, often termed "local arthritis deformans."

He groups normal joints according to their relations with neighboring joints into static (mechano- functinal) unities in which a static disturbance of one joint in the unity makes itself felt more or less in all the rest. The most important feature of such disturbances, is an alteration of the normal opposition of cartilaginous joint surfaces, throwing certain surfaces or parts of them, permanently out of function leaving the rest of the joint insufficient for the strains of ordinary use and setting up a train of compensatory growth phenomena, leading to exostoses, and to outgrowth from the soft parts.

This change in the normal contact of joint surfaces he terms joint surface incongruence ("Gelenkflächenin-congruenz.")

The author has made interesting observations on the normal relation of the femur to the pelvis. He finds that the normal relation of the joint of the femur to the pelvis varies in an important degree as it affects its function, not according to the angle of the neck to the shaft, nor to the shape of he acetabulum nor to its position above or below Nelaton's line as heretofore considered. He essays to demonstrate that the important relation is in the anterior, median or posterior position of the acetabulum. The median position he considers normal, the anterior or posterior abnormal and responsible in large measure for changes leading to static defects in the lower limbs and therefore to pathologic disturbances

Among other theories he argues convincingly that the so called "malum coxae senile" is simply a late (very late) stage of "arthritis deformans coxae" the first result of the joint surface incongruence entailed.

It is impossible adequately to summarize, much less discuss, this monograph in the allotted space, in view of the many novel conceptions supported by a vast amount of collected and tabulated measurements and X-ray plates, with a large assortment of case histories in point. The systematic arrangement of the work as a whole offsets the author's cumbrous German style, as he frequently attains orotundity at the expense of lucidity and precision.

The author might also be criticised for an apparent neglect of the foreign literature. He does not cite a single article, outside of the German periodicals. When he laments the absence of literature or the sacro-iliac joint we are inclined to feel that he has slighted American writers, notably Goldthwaite.

J. S. LEWIS.

